# FINANCIAL HEALTH AND WELL-BEING OF RURAL CAREGIVERS OF OLDER ADULTS WITH CHRONIC ILLNESSES

**DOI:** 10.1093/geroni/igae098.3983

**Published:** 2024-12-31

**Authors:** Evans Osei, Nasreen Lalani, Siqi Yang, Sampada Wagle, Bhagyashree Katare

**Affiliations:** Purdue University, West Lafayette, Indiana, United States; Purdue University, West Lafayette, Indiana, United States; Purdue University, West Lafayette, Indiana, United States; Purdue University, West Lafayette, Indiana, United States; Purdue University, West Lafayette, Indiana, United States

## Abstract

Rural caregivers experience various financial stressors and challenges in providing care to their ill family members. Rural health disparities such as limited access to care, economic opportunities, resources, and support pose increased financial threats and insecurity resulting in poor patient and caregiver outcomes. Our study aimed to explore perceived financial stressors/challenges and their impacts on the self-care and well-being of rural caregivers of older family members with serious chronic illness. Using a purposive sampling approach, we did qualitative interviews among N=20 rural female caregivers caring for their older family member with any serious chronic illness. Interviews were done in-person and online, interview duration was about 30-45 minutes. Data was transcribed and analyzed deductively and inductively using thematic content analysis. Major financial impacts of caregiving included: leaving job or reducing work hours, time constraints on caregivers’ self-care, inability to balance family relations and caregiving roles, and difficulty navigating financial processes in the existing healthcare system. These challenges led to cutting down on groceries, reliance on retirement savings, and lack of personal time. Participants faced sleep and diet disruptions, shouldered financial decisions alone with limited or no support, and felt guilty over unfulfilled care recipient wishes due to financial constraints. Adequate self-care, coping, and well-being are associated with improved financial well-being and reduced financial threat. Caregiver support programs should focus on strategies and interventions to improve the financial well-being and overall quality of life of caregivers in the rural communities.

